# Persistent Trigeminal Artery Playing a Protective Role in a Case of Vertebral Artery Dissection and Stenosis

**DOI:** 10.7759/cureus.5327

**Published:** 2019-08-05

**Authors:** Michelle Nunes, Aqsa Ullah, Joseph Rios, Ankur Garg

**Affiliations:** 1 Neurology, University of Central Florida/Osceola Regional Medical Center, Orlando, USA; 2 Vascular Neurology, Orlando Neurosurgery, Winter Park, USA

**Keywords:** ischemic stroke, anatomical variations, vascular anatomy, persistent primitive trigeminal artery, vertebral artery, neurointerventional procedures, vertebral artery dissection, neuroradiology, neurological symptoms, headaches

## Abstract

Several fetal anastomoses have been described between the carotid and vertebrobasilar circulations. These anastomoses usually revert while the P1 segment (posterior cerebral artery segment 1) develops. However, these primitive intracranial embryonic anastomes can occasionally persist in adult age. Persistence of the primitive trigeminal artery (PPTA) is the most common of these persistent fetal anastomoses. Although uncommonly identified, knowledge of this structure is crucial for clinicians who analyze cranial imaging. PPTA has been associated with intracranial aneurysms and arteriovenous malformations but it is not very clear whether or not PPTA can also play a protective role in certain cases. We present the case of a 31-year-old female who suffered from a medullary stroke due to vertebral artery dissection and the persistent trigeminal artery (PTA) possibly played an important protective role since blood flow to the brainstem was preserved via a robust collateral flow from the right internal carotid artery (ICA).

## Introduction

Several fetal anastomoses have been described between the carotid and vertebrobasilar circulations [1]. Hypoglossal, trigeminal, otic, and proatlantal arteries are examples of variant anatomical arterial communications between the anterior and posterior circulation [1]. These anastomoses usually revert while the posterior cerebral artery segment 1 (P1) segment develops [2]. However, these primitive intracranial embryonic anastomoses can occasionally persist in adulthood. Persistence of the primitive trigeminal artery (PPTA) is the most common of these persistent fetal anastomoses. The prevalence is between 0.1 to 0.68% [2].^ ^In utero*,* the persistent trigeminal artery (PTA) supplies the basilar artery before the development of the posterior communicating and vertebral arteries [1]. The PTA arises from the junction between the petrous and the cavernous segment of the internal carotid artery (ICA) and runs posterolaterally along the trigeminal nerve or crosses over or through the dorsum sellae [3]. 

Although uncommonly identified, knowledge of this structure is crucial for clinicians who analyze cranial imaging, such as a neurologist, as well as for those who perform invasive studies and operate in this region, such as neurosurgeons and interventional neurologists. An increased occurrence of other coexisting intracranial vascular abnormalities has been reported in patients with PPTA. The clinical relevance of a PTA is debatable as most cases are discovered incidentally [2]. Herein, we present a case where the presence of a PTA played a protective role in a patient with a vertebral artery dissection. 

## Case presentation

**Figure 1:**
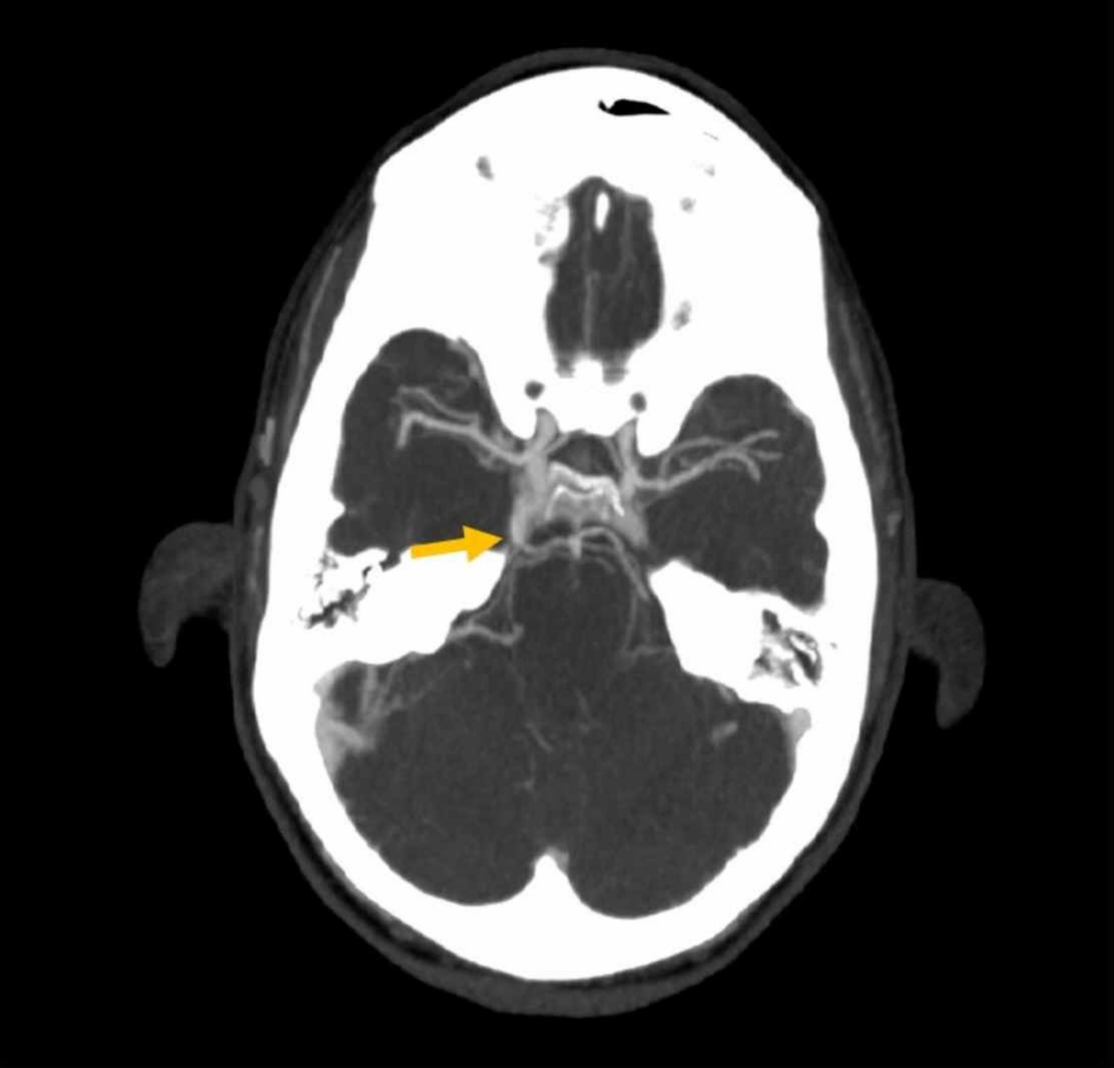
Axial CT angiography of the brain Image displays atypical vessel coursing from the cavernous right internal carotid artery (ICA) to the basilar artery within the prepontine cistern.

**Figure 2:**
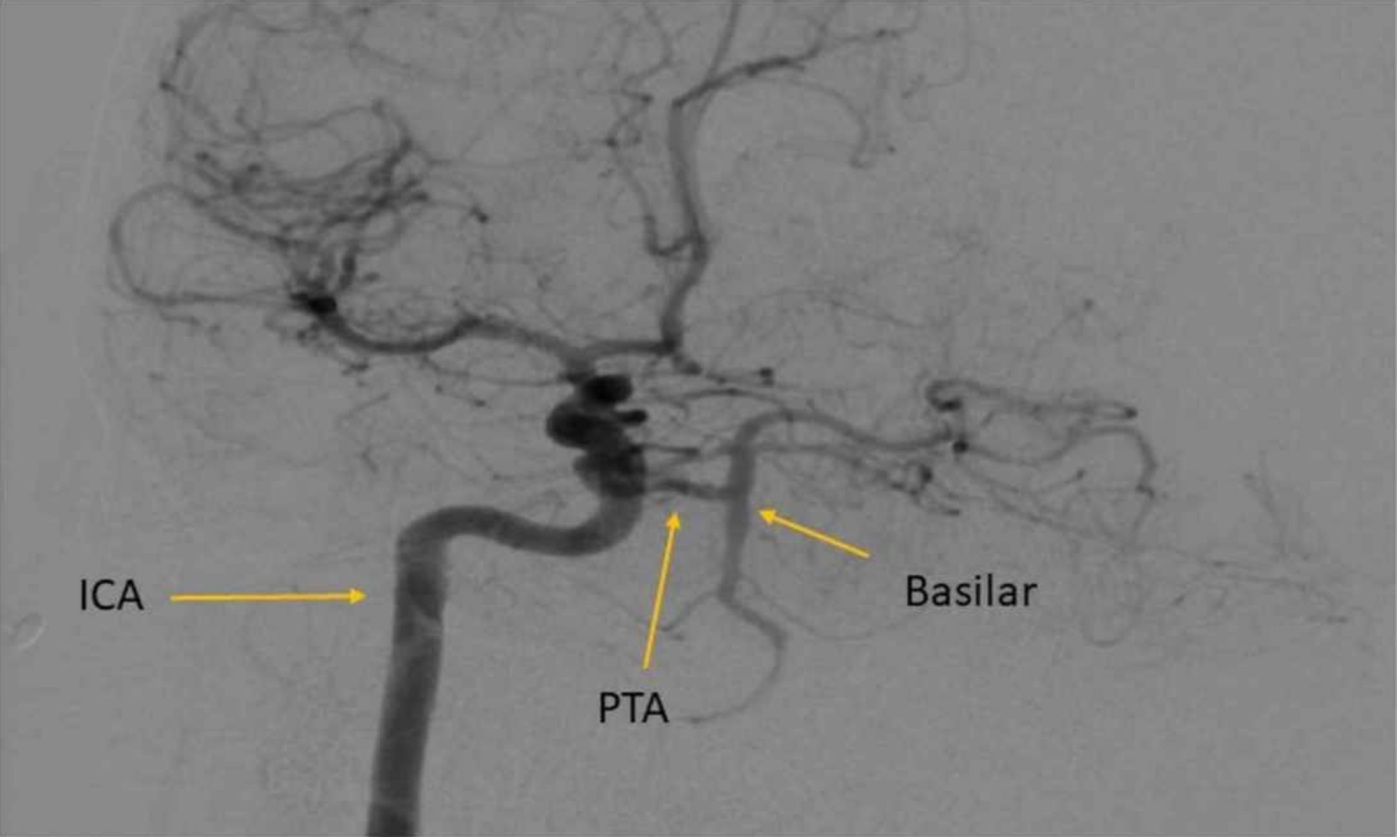
Oblique digital subtraction angiography (DSA) of the right internal carotid artery (ICA) Angiography demonstrates an aberrant vessel communicating between the cavernous segment of the ICA with the basilar artery consistent with persistent trigeminal artery (PTA).

A previously healthy 31-year-old, right-handed woman presented to the emergency department (ED) due to the sudden onset of right facial numbness, followed by left arm and leg numbness and weakness. Three days prior to presentation, she had visited a chiropractor for evaluation of neck pain that had resulted after a mild neck injury while playing football that same week. A high-velocity chiropractic technique was applied to her neck with subsequent relief of the neck pain. Three days later, however, she woke up with right neck pain, right-sided headache, and right facial numbness, which was shortly followed by weakness and numbness of the left upper and lower extremities. Upon arrival to the ED, the patient’s symptoms had mostly improved with only mild residual headache, right neck pain, and right facial numbness. The National Institutes of Health Stroke Scale (NIHSS) score was 1 due to a decreased pinprick sensation on the right face. Initial head computed tomography (CT) was negative for hemorrhage; however, computed tomography angiography (CTA) showed a right vertebral artery dissection with high-grade stenosis at the V2/V3 segments and an atypical vessel coursing from the right ICA to the basilar artery, suspicious for a PTA (Figure 1). Stroke protocol magnetic resonance imaging (MRI) revealed a small right lateral medullary infarct. She was treated with a continuous intravenous heparin drip. The following morning, she experienced recurrence of her initial symptoms and was, therefore, taken for digital subtraction angiography (DSA). Angiography confirmed a Grade II dissection of the right vertebral artery at the distal V2 and V3 segments with associated critical stenosis and a fetal variant posterior cerebral artery (PCA). Interestingly, a large PTA was noted that provided a robust collateral flow from the cavernous segment of the right internal carotid artery to the posterior cerebral circulation (Figure 2). 

## Discussion

PTA is a rare remnant of the embryonic circulatory system that connects the intracavernous segment of the ICA with the middle or distal portion of the basilar artery. Although uncommonly identified in the general population, PTA is the most common persistent fetal anastomosis described [2]. At 28 to 29 days of embryonic development, the ICA starts supplying the forebrain and hindbrain. By this time, the trigeminal artery is observed at the level of the trigeminal ganglion^ ^providing vascular supply to the brainstem structures [1]. The anastomoses between the ICA and posterior brain circulation exists for only four to eight days before disappearing as the basilar artery (BA) ends its formation,^ ^with thePTA usually regressing at the carotid edge [1-4]. Persistence of this primitive intracranial embryonic anastomosis can occur. Such persistent anastomosis has an unknown etiology and different clinical implications [1-6]. 

The vast majority of PTA cases described in the literature were incidentally found on imaging performed for unrelated reasons; therefore, clinical implication is uncertain [2]. PTA has been associated with intracranial aneurysms, arteriovenous malformations, carotid-cavernous fistulas, trigeminal neuralgia, and cerebellar hemangioblastoma [1-6]. Also of relevant importance is the fact that PTA is frequently associated with BA hypoplasia, and in this condition, the majority of the blood flow to the upper pons, mesencephalon, cerebellum, and basal surfaces of the temporal and occipital lobes is provided by the ICA via the PTA [2]. In these cases, posterior circulation strokes have been reported secondary to dissection, atherosclerotic lesions, and cardiac emboli of the ICA [2]. In our case, however, we believe that the existence of a PPTA played an important protective role. Our patient has a Grade II vertebral artery dissection with severe stenosis which could have provoked a catastrophic brainstem ischemic stroke. Yet, since blood flow to the brainstem was preserved via a robust collateral flow from the right ICA, she suffered a mild medullary stroke with minimal neurological consequences. 

Moreover, during the planning of surgical approaches to the skull base, it is important to evaluate the vascular anatomy of the sellar and parasellar regions, searching for PTA. The unnoticed presence of a PTA may result in disastrous outcomes following approaches to the sellar or parasellar regions, the cavernous sinus, or Gasserian ganglion [2]. 

## Conclusions

Persistence of fetal anastomosis are rare but of significant importance for clinicians who analyze brain imaging, as well as for neurosurgeons and neurointerventionalists as they perform invasive studies and operate on this region. 

Clinical significance of the existence of a persistent trigeminal artery is uncertain. PTA has been implicated with posterior circulation strokes and other vascular malformations, such as an aneurysm, arteriovenous malformations (AVMs), and trigeminal neuralgia. We believe that in cases of vascular lesions of the posterior circulation, such as vertebral artery dissection, having a connection between the anterior and posterior circulation systems may have a protective role since blood flow will still be preserved. 
